# Retraction Note: Optimization of off-grid hybrid renewable energy systems for cost-effective and reliable power supply in Gaita Selassie Ethiopia

**DOI:** 10.1038/s41598-026-42629-2

**Published:** 2026-03-03

**Authors:** Elsabet Ferede Agajie, Takele Ferede Agajie, Isaac Amoussou, Armand Fopah-Lele, Wirnkar Basil Nsanyuy, Baseem Khan, Mohit Bajaj, Ievgen Zaitsev, Emmanuel Tanyi

**Affiliations:** 1 Department of Electrical and Electronic Engineering, Faculty of Engineering and Technology, University of Buea, PO. Box. 63, Buea, Cameroon; 2 Department of Electrical and Computer Engineering, Debre Markos University, Debre Markos, Ethiopia; 3 Department of Mechanical Engineering, Faculty of Engineering and Technology, University of Buea, PO. Box. 63, Buea, Cameroon; 4 Department of Electrical and Computer Engineering, Hawassa University, Hawassa, Ethiopia; 5 Department of Electrical Engineering, Graphic Era (Deemed to be University), Dehradun, 248002 India; 6 Hourani Center for Applied Scientific Research, Al-Ahliyya Amman University, Amman, Jordan; 7 Graphic Era Hill University, Dehradun, 248002 India; 8 Department of Theoretical Electrical Engineering and Diagnostics of Electrical Equipment, Institute of Electrodynamics, National Academy of Sciences of Ukraine, 56, Peremogy, Kyiv-57 03680 Ukraine; 9 Center for Information-Analytical and Technical Support of Nuclear Power Facilities Monitoring of the National Academy of Sciences of Ukraine, Akademika Palladina Avenue, 34-A, Kyiv, Ukraine

Retraction of: *Scientific Reports* 10.1038/s41598-024-61783-z, published online 13 May 2024

The Editors have retracted this Article.

Following publication, concerns were raised regarding the inclusion of irrelevant references. Further investigation by the Editors revealed inconsistencies between the text and at least 30 cited references, and the authorship of the Article could not be verified. Upon request, the Authors could not provide a satisfactory response to the concerns raised. The Editors therefore no longer have confidence in the reliability of the content of this Article.

Mohit Bajaj and Ievgen Zaitsev do not agree with this retraction. Elsabet Ferede Agajie, Takele Ferede Agajie, and Baseem Khan did not explicitly state if they agree or disagree with the retraction. Isaac Amoussou, Armand Fopah-Lele, Wirnkar Basil Nsanyuy, and Emmanuel Tanyi did not respond to the Editors’ communication about this retraction.

